# Diseases associated with prematurity in correlation with N-terminal pro-brain natriuretic peptide levels during the early postnatal life

**DOI:** 10.1007/s00431-023-04973-7

**Published:** 2023-04-18

**Authors:** Agnes-Sophie Fritz, Titus Keller, Angela Kribs, Christoph Hünseler

**Affiliations:** Neonatal Intensive Care Unit, University Hospital in Cologne, Kerpener Str. 34, 50937 Cologne, Germany

**Keywords:** N-terminal pro-brain natriuretic peptide, Preterm infant, Patent ductus arteriosus, Bronchopulmonary dysplasia, Pulmonary hypertension

## Abstract

The aim of this observational study was to investigate the influence of different typical preterm diseases on NT-proBNP serum levels in the early postnatal period of life of a preterm infant. NT-proBNP levels of 118 preterm infants born ≤ 31 weeks GA were determined at the first week of life, after 4 ± 1 weeks of life, and at a corrected gestational age of 36 + 2 weeks. Relevant complications with a possible influence on NT-proBNP values in the first week of life such as early neonatal infection, hemodynamically significant PDA (hsPDA), early pulmonary hypertension (early PH), and intraventricular hemorrhage (IVH) were evaluated; at 4 ± 1 weeks of life, bronchopulmonary dysplasia (BPD), BPD-related pulmonary hypertension (BPD-associated PH), late infection, IVH, and intestinal complications were evaluated. At a corrected gestational age of 36 ± 2 weeks, we examined the effect of retinopathy of prematurity (ROP), BPD, BPD-associated PH, and late infection on NT-proBNP levels. In the first days of life, only the isolated occurrence of hsPDA resulted in significantly increased NT-proBNP levels. In multiple linear regression analysis, early infection remained independently associated with NT-proBNP levels. At 4 ± 1 weeks of age, the isolated presence of BPD and BPD-related PH resulted in increased levels, and the effect remained significant in the multiple regression analysis. At a corrected gestational age of 36 ± 2 weeks, infants with relevant complications at this final evaluation time tended to have lower NT-proBNP values than our exploratory reference values.

*Conlusion*: NT-proBNP in the first week of life seems to be mainly influenced by an hsPDA and infection or inflammation. BPD and BPD-related PH are the most important factors influencing NT-proBNP serum levels in the first month of life. When preterm infants reach a corrected GA of 36 ± 2 weeks, chronological age rather than complications of prematurity must be considered when interpreting NT-proBNP levels.

**Table:** 

**What is Known:** *• Several complications associated with prematurity, such as hemodynamically significant PDA, pulmonary hypertension, bronchopulmonary dysplasia, and retinopathy of prematurity, have been shown to influence NT-proBNP levels in preterm infants in their early postnatal life.*	
**What is New:** *• Hemodynamically relevant PDA is a major factor in the increase of NT-proBNP levels in the first week of life.* *• Bronchopulmonary dysplasia and pulmonary hypertension associated with bronchopulmonary dysplasia are important factors in the increase in NT-proBNP levels in preterm infants at approximately 1 month of age.*

**What is Known:**

*• Several complications associated with prematurity, such as hemodynamically significant PDA, pulmonary hypertension, bronchopulmonary dysplasia, and retinopathy of prematurity, have been shown to influence NT-proBNP levels in preterm infants in their early postnatal life.*

**What is New:**

*• Hemodynamically relevant PDA is a major factor in the increase of NT-proBNP levels in the first week of life.*

*• Bronchopulmonary dysplasia and pulmonary hypertension associated with bronchopulmonary dysplasia are important factors in the increase in NT-proBNP levels in preterm infants at approximately 1 month of age.*

## Introduction

Brain natriuretic peptide (BNP) is a peptide hormone that regulates volume expansion or pressure overload through increased diuresis and natriuresis, vasodilation and by inhibiting the production of renin and aldosterone [1]. Cardiomyocytes of the ventricles of the heart secrete an inactive prohormone, pro-BNP, that is cleaved at a ratio of 1:1 into the biologically active BNP and the inactive N-terminal pro BNP (NT-proBNP) [2]. NT-proBNP proved to have more stability in vitro and a longer half-life than BNP (120 min vs. 22 min) [3]. It thus appears in a higher plasma concentration than the actual hormone BNP. NT-proBNP is dependent on renal function which must be taken into account when interpretating serum levels [4].

BNP is well known as a diagnostic marker of congestive heart failure in adults [5]. In preterm neonates, increased NT-proBNP and BNP values have been associated with the following diseases of prematurity: hemodynamically significant patent ductus arteriosus (hsPDA) [6, 7], pulmonary hypertension (PH) [8], bronchopulmonary dysplasia (BPD) [9], retinopathy of prematurity (ROP) [10, 11], inflammation or sepsis [12, 13]and congenital heart diseases [14]. Still, how the effect of the different diseases upon NT-proBNP must be valued remains inconclusive.

The aim of this retrospective observational analysis was to evaluate if NT-proBNP is a reliable parameter for the diagnosis of diseases related to prematurity. Additionally, we aimed to estimate the influence of the different complications on NT-proBNP values in comparison to each other depending on postnatal age.

## Materials and methods

The data of 118 preterm infants ≤ 31 weeks GA treated between October 1, 2017, and June 30, 2019, in the Neonatal Intensive Care Unit (NICU) of the Children’s Hospital of the University Hospital in Cologne, Germany, were included. Approval by the local ethics committee was obtained previously. Parental consent was not required as NT-proBNP determination is part of routine blood collections at our institution. According to our institutional protocol, the first week of life, around day 28 of life, and when infants reach a corrected GA of 36 weeks define the timing periods for NT-proBNP measurements. Actual timing of NT-proBNP measurements partly differ from the institutional protocol and the following three sampling times were identified: day 2 to 11 of life (in the following called: first week of life), day 18 to 36 of life (4 ± 1 weeks of life), and when the preterm infants reached a corrected GA of 34 + 3 to 40 + 3 weeks (corrected GA of 36 ± 2 weeks). The higher NT-proBNP value was chosen if multiple samples from the same preterm infant were available for a sampling time. Missing values emerged randomly or were due to a condition of the neonate that did not allow blood sampling, a potential source of bias. Arterial, central or peripheral venous, or capillary blood samples were analyzed on the Cobas E801 analyzer using the Elecsys proBNP II method (Roche Diagnostics). Different types of blood sampling are known to lead to comparable NT-proBNP values [15]. NT-proBNP values between 5 to 35,000 ng/l can be measured with the used test. Values above the upper measuring limit are given as 35,000 ng/l or up to 70,000 ng/l for twofold diluted samples. The intra-assay coefficient of variation for human serum varied between 1.5 and 9.2%, and the interassay coefficient of variation for human serum between 2.6 and 12.5% [16].

**Fig. 1:**
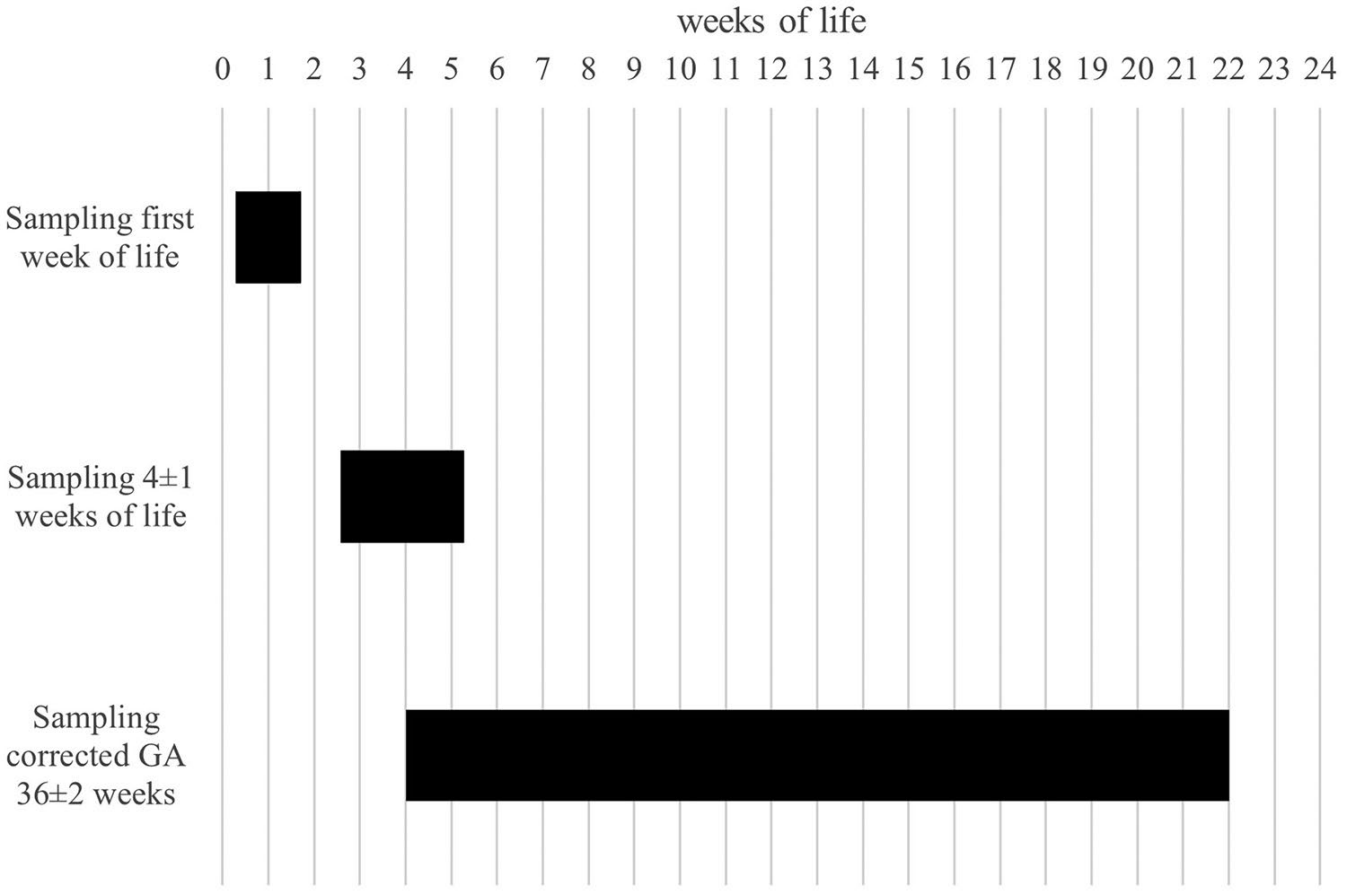
Sampling times according to weeks of life

Originally designed as a longitudinal study design, the composition of groups partly differs over time resulting in characteristics of a cross-sectional study. Figure 1 shows the different sampling times according to the postnatal age of the preterm infants. The samplings at 4 ± 1 weeks of life and at a corrected GA of 36 ± 2 weeks partly overlap as in less premature infants, the chronological age of around 4 weeks possibly coincidences with the corrected GA of 36 weeks.

The following criteria define the different complications:

(I) Infection: elevated laboratory values (interleukin-6 > 50 ng/l, C-reactive protein (CRP) > 5 mg/l, leucocytes > 20,000/µl or left shift) or clinical signs of infection with subsequent antibiotic treatment, occurring during the first 72 h of life (early infection) or thereafter (late infection)
(II) Hemodynamically significant PDA: two of the following criteria were observed by echocardiography: 1. diameter of the ductus arteriosus: ≥ 2.0 mm, 2. degree of pulmonary overflow: left atrial to aortic root ratio (La:Ao) ≥ 1.4, 3. retrograde descending aortic flow, 4. organ blood flow: low antegrade flow in systole or diastole or absent/reversed diastolic flow
(III) BPD: definition according to Jobe [17]
(IV) Pulmonary hypertension: defined by (1) pulmonary arterial pressure systolic (PAPs)/ systolic systemic pressure ratio > 0.5 (PAPs was estimated by the right ventricular pressure which was calculated by tricuspid regurgitation jet velocity), (2) interventricular septum-configuration: flat or bowed to the left and (3) right to left or bidirectional shunting at the patent foramen ovale or PDA,diagnosed within the first 7 days of life (early pulmonary hypertension) or thereafter (BPD related pulmonary hypertension [18])
(V) ROP Stage 2 with Plus disease or higher: definition according to the international definition of ROP [19]
(VI) Intraventricular hemorrhage (IVH) grade 2 or higher: definition according to Papile [20]
(VII) Intestinal complications, e.g. all stages of necrotizing enterocolitis (NEC) according to the modified Bell staging criteria [21], focal intestinal perforation (FIP) or gastric perforation.

Major congenital heart diseases, chromosomal abnormalities, or kidney diseases defined exclusion criteria.

In an earlier published work, we described exploratory NT-proBNP reference values, based on the values of premature infants ≤ 31 weeks GA that proved to have no relevant complications during their early postnatal life [20]. The present analysis focuses on the different diseases of prematurity and their relationship with NT-proBNP values. In a first step, we assigned the different complications of prematurity to the three different sampling times, depending on their time of occurrence and clinical relevance. Secondly, we compared the NT-proBNP values of the preterm infants showing a certain complication to the exploratory reference values of our recent publication [20]. In a third step, we compared the NT-proBNP values of the preterm infants that proved to have only one relevant complication for the time period to our reference values. In a concluding multiple linear regression, the effect of the different diseases of prematurity and certain baseline characteristics upon NT-proBNP values was analyzed for each sampling time.

## Statistical analysis

Statistical analysis was performed using the Statistical Package for Social Sciences, v27 (IBM SPSS, Chicago, Ill., USA). Mann–Whitney-U-Test and Kruskal–Wallis-Test were used to compare NT-proBNP concentrations between two or more groups. In multiple linear regression analysis, diseases of prematurity represented independent variables that aimed to explain the dependent variable NT-proBNP values at the different sampling times. Multicollinearity was investigated through Pearson’s correlation coefficient [22] and variance inflation factor [23]; multicollinearity was stated for a correlation coefficient above 0.7 and variance inflation factor above 10. The entry method was the chosen variable selection method. Goodness of fit was detected by using the coefficient of multiple determination (R^2^) which is the proportion of variation in the dependent variable that can be explained by the multiple regression model based on the independent variables. The coefficient of multiple determination was classified according to Cohen [24]; R^2^ ≤ 0.02 was indicative for a poor, R^2^ ≤ 0.13 as a moderate and R^2^ ≤ 0.26 as a high goodness-of-fit. The independent variables for multivariate analysis at each sampling time were chosen if there was strong study-based evidence for their relevance for NT-proBNP values.

## Results

### Patient cohort

Characteristics and postnatal course of 118 patients are described in Table 1. The median GA at birth was 26 + 5 weeks and the median birth weight 900 g. One patient died after 5 days because of severe IVH. It is important to emphasize that group numbers in Table 1 differ from the group numbers in univariate and multivariate analysis. In Table 1 the characteristics of all patients of the study are described whereas in univariate and multivariate analysis the subgroups of patients with disponible NT-proBNP serum levels at the sampling time are analyzed.

**Table 1:** Characteristics and postnatal course of patients

**Characteristics**	All Infants: n = 118 number (percentage) or median (IQR)
Gender, male	69 (59%)
Gestational age at birth (weeks)	26 + 5 (24 + 5–29 + 2)
Birth weight (g)	900 (630–1,220)
SGA (birth weight < 3^rd^ percentile)	9 (8%)
5’ Apgar score	7 (7–8)
CRIB-II-score	5 (2–9)
C-section	108 (92%)
Prenatal steroids	90 (76%)
▪ Complete	67 (57%)
▪ Incomplete	23 (19%)
Early infection	14 (12%)
Late infection	25 (21%)
PDA	94 (80%)
▪ non-hemodynamically significant	76 (64%)
▪ hemodynamically significant	18 (15%)
Early Pulmonary Hypertension	11 (9%)
BPD related Pulmonary Hypertension	17 (14%)
BPD	37 (31%)
▪ mild	26 (22%)
▪ moderate	6 (5%)
▪ severe	5 (4%)
ROP	101 (86%)
▪ Stage 1	79 (67%)
▪ Stage 2	12 (10%)
▪ Stage 3	10 (8%)
IVH	37 (31%)
▪ Grade 1	23 (19%)
▪ Grade 2	3 (3%)
▪ Grade 3	11 (9%)
Intestinal complications	15 (13%)
▪ NEC	4 (3%)
▪ FIP	9 (8%)
▪ Gastric perforation	2 (2%)
Pulmonary complications (pneumothorax, pulmonary interstitial emphysema)	10 (8%)
Duration of ventilation (mechanical ventilation and CPAP) (hours)	835 (231–1564)
Duration of oxygen supply > 21% (days)	3 (1–46)

## Results

Preterm infant diseases in correlation with NT-proBNP levels at the different collection time points.

### First week of life

Univariate analysis: we selected the following conditions associated with preterm birth as relevant to the first week of life: hsPDA, early infection, primary PH, and IVH > grade 1.

As a first step, we investigated whether preterm infants with each complication had higher serum NT-proBNP levels compared with our exploratory reference values. Our results showed that preterm infants with hsPDA (n = 8, median: 7843 ng/l, IQR: 2915–14,116 ng/l; p = 0.004), early infections (n = 9, median: 9654, IQR: 2356–33,530; p = 0.009), primary PH (n = 8, median: 7843, IQR: 2163–26,493; p = 0. 022) or IVH > grade 1 (n = 9, median: 5508, IQR: 3611–23,019; p = 0.003) had significantly higher NT-proBNP values at the first week of life compared to the reference group (n = 27, median: 1896, IQR: 1277–5200). To specify the influence of a single complication on NT-proBNP levels, we investigated whether the isolated presence of the complication also resulted in increased NT-proBNP levels. Indeed, the isolated presence of the relevant complications in the first week of life resulted in higher NT-proBNP levels, but the difference was statistically significant only in the preterm infants with the isolated presence of hsPDA (n = 4, median: 6483, IQR: 2839–14,116, p = 0.046).

**Fig. 2:**
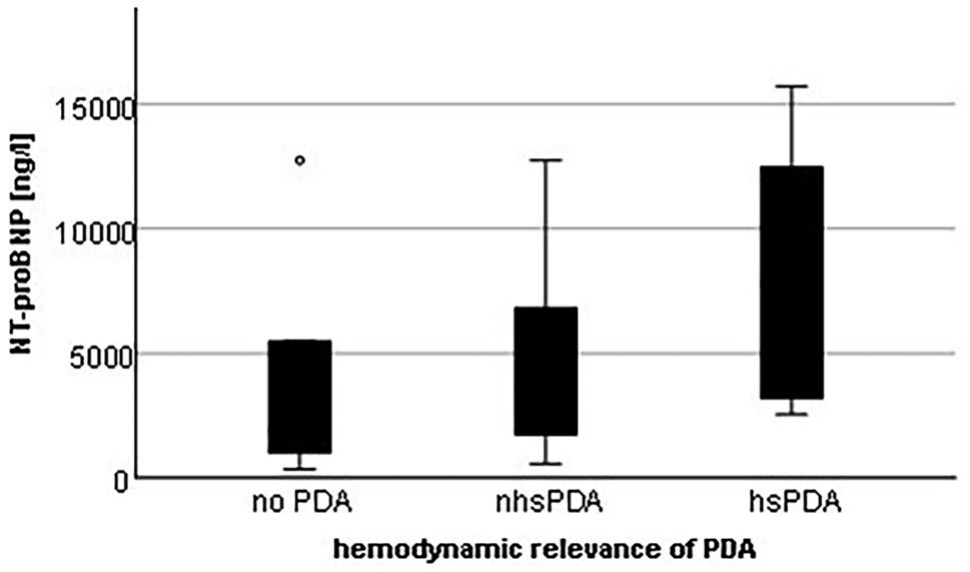
NT-proBNP levels in correlation with the hemodynamic relevance of PDA

When comparing all three PDA grades of preterm infants with available NT-proBNP of the first week of life (no PDA: n = 17, nhsPDA: n = 36 and hsPDA: n = 8), NT-proBNP serum levels appeared to increase with hemodynamic relevance of PDA in the first week of life, but differences in NT-proBNP levels did not reach statistical significance (Fig. 2).

Multivariate analysis: multiple linear regression included the following variables: Gestational age at birth, PDA status, early infection, and primary PH. Data from 61 patients were analyzed. Multicollinearity between independent variables was excluded. The R^2^ for the overall model was 0.347 (adjusted R^2^ = 0.300), indicating a high goodness of fit.Only the variable early infection (regression coefficient = 11,898, p < 0.001) proved to have an independent effect on NT-proBNP values at the first week of life.

### 4 ± 1 weeks of life

Univariate analysis: we correlated NT-proBNP levels at 4 ± 1 weeks of life with BPD, BPD-related PH, late infections, intestinal complications and IVH. Preterm infants with the above complications had significantly higher NT-proBNP levels compared to the reference group (BPD: n = 26, median: 1186 ng/l, IQR: 813–2634 ng/l, p < 0.001; BPD-related PH: n = 13, median: 1686, IQR: 823–3117, p < 0.001; late infection: n = 17, median: 805, IQR: 538–1462, p < 0.001; intestinal complications: n = 8, median: 1953, IQR: 959–3368, p < 0.001; IVH: n = 7, median: 2,446, IQR: 1089–3335, p < 0.001 vs. comparison group: n = 26, median: 463, IQR: 364–704). Moreover, preterm infants with the isolated presence of BPD (n = 6, median: 869, IQR: 487–1138) or the isolated presence of BPD-related PH (n = 5, median: 894, IQR: 823–2176) had significantly higher NT-proBNP values compared to our reference values (p = 0.029 and p < 0.001).

**Fig. 3:**
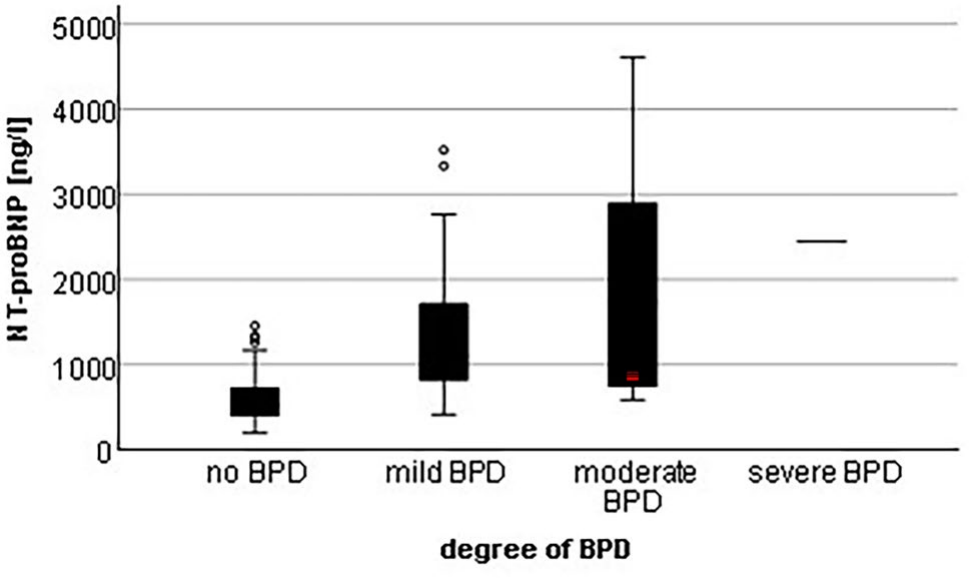
NT-proBNP levels in correlation with the degree of BPD

When comparing all 4 grades of BPD of preterm infants with existing NT-proBNP values at 4 ± 1 weeks of life (no BPD: n = 45, mild BPD: n = 19, moderate BPD: n = 6 and severe BPD: n = 1), NT-proBNP values were significantly higher in infants with mild or moderate BPD than in infants without BPD (p < 0.001 and p = 0.031). A trend of increasing NT-proBNP values with increasing severity of BPD was observed (Fig. 3).

Multivariate analysis: multiple linear regression included the following variables: BPD, BPD-related PH, and late infection. One outlier was excluded, resulting in a group size of n = 70. Multicollinearity between independent variables was excluded. The R^2^ for the overall model was 0.0382 (adjusted R^2^ = 0.353), indicating a high goodness of fit. The variables BPD (regression coefficient = 573, p = 0.005) and BPD-related PH (regression coefficient = 642, p = 0.011) were found to independently influence NT-proBNP values at 4 ± 1 weeks of age.

### Corrected GA of 36 ± 2 weeks

Univariate analysis: we considered BPD, BPD-related PH, ROP stage 2 with plus disease or higher, and late infections as diseases of preterm infants relevant to the sampling time. The NT-proBNP levels of the preterm infants who had the above complications were not elevated compared with the NT-proBNP reference values (BPD: n = 16, median: 656 ng/l, IQR: 408–879 ng/l; BPD-related PH: n = 5, median: 469, IQR: 332–941; ROP stage 2 with plus disease or higher: n = 4, median: 645, IQR: 377–1567 vs. Reference group: n = 33, median: 824, IQR: 714–1232). In contrast, reference NT-proBNP values were significantly higher than NT-proBNP values of infants with BPD (p = 0.021) and higher than NT-proBNP values of infants with isolated presence of BPD (n = 4, median: 568, IQR: 316–661, p = .009).

## Discussion

The aim of the present analysis was to investigate the relevance of NT-proBNP as a diagnostic marker for conditions associated with preterm birth in preterm infants with a gestational age of ≤ 31 weeks in routine clinical practice.

There is strong evidence that various preterm conditions affect NT-proBNP serum levels, including hemodynamically significant patent ductus arteriosus [6, 7], pulmonary hypertension, bronchopulmonary dysplasia [9], retinopathy of prematurity [10, 11], and inflammation or sepsis [12, 13].For other preterm diseases such as intestinal complications (NEC, FIP, gastric perforation) and IVH, the study evidence on their effects on NT-proBNP levels is less clear. We consequently excluded those two complications from our multivariate analysis. However, it is likely that hemodynamic changes due to increased intra-abdominal pressure, volume depletion, and inflammatory processes also affect NT-proBNP levels in these intestinal complications. We additionally included the independent variable gestational age in the multivariate analysis of the first week of life, as gestational age proved to have an independent influence on NT-proBNP values in our previous study on this topic [25]. Complications regularly and frequently occur simultaneously in preterm infants ≤ 31 weeks GA. Studies on how severe to assess the impact of a single complication are lacking so far.

Our results show that all relevant complications lead to an increase in NT-proBNP serum levels in the first week of life. In a further analysis to specify the effects, we found that the infants with the isolated presence of hsPDA had significantly higher NT-proBNP levels compared with the reference levels. In multiple linear regression, early infection was found to be independently associated with NT-proBNP levels.

The significance of hsPDA on NT-proBNP serum levels in the first days of life is less surprising and is consistent with other studies [6, 7]. Also, the correlation between increasing hemodynamic relevance of PDA status and increasing NT-proBNP levels, as observed in our study, has been described previously [6, 26]. Nevertheless, it is surprising that early infection, but not the variable hsPDA, remained independently associated with NT-proBNP levels in the multiple regression analysis. There are several explanations for this: a. GA at birth, as a relevant factor for NT-proBNP levels in the first week of life, was additionally included in the multiple regression analysis, possibly weakening the significance of the other variables in the analysis. b. NT-proBNP values measured in the first week are extremely high and widely scattered. In particular, the values of infants with early infection have a wide range and include the highest measured NT-proBNP value in our study (39,340 ng/l), possibly leading to a false dominance of the variable. On the other hand, the influence of early infection on NT-proBNP levels may have been underestimated in recent studies, and hemodynamic changes and direct stimulation of BNP production by stimulatory cytokines may play a much more important role than expected [12, 27].

Our analysis of NT-proBNP levels at 4 ± 1 weeks of age showed that the isolated presence of BPD or the isolated presence of BPD-related PH resulted in increased NT-proBNP levels compared with reference values. Multiple regression analysis confirmed the independent association of BPD and BPD-related PH with serum NT-proBNP levels.

Analogous to the correlation between NT-proBNP levels at the first week of life and the severity of PDA status, a tendency for NT-proBNP levels to increase at 4 ± 1 weeks of life with increasing degree of BPD was observed.

That BPD and BPD-associated PH associated with preterm birth are reflected in NT-proBNP levels is consistent with other studies on this topic [8, 9, 28]. Pathophysiologically, both conditions are thought to stimulate NT-proBNP production through increased volume and pressure stress caused by increased mean pulmonary artery pressure (PH) or alveolar growth restriction with consequent reduced pulmonary vascular growth (BPD).

Regarding NT-proBNP values at 36 ± 2 weeks of age with corrected GA, our reference NT-proBNP values tended to be higher than NT-proBNP values of preterm infants with complications. We have described this phenomenon in our previous study of exploratory NT-proBNP reference values in preterm infants [25]. It is well known that NT-proBNP serum levels steadily decrease with increasing chronological age [29].

Our NT-proBNP reference values are based on NT-proBNP serum levels in preterm infants without relevant complications due to their prematurity. They tend to have a higher GA at birth and consequently a lower chronological age when they reach a corrected gestational age of 36 ± 2 weeks, which is a possible explanation for the discrepancy in values at this time point.

## Limitations of the study

An important limitation of the study is the definition of the pulmonary hypertension. In the best case scenario, the definition of the pulmonary hypertension should have been based on the oxygenation index together with the measured PAPs, like demonstrated by Piastra et al. [30]. Such a definition could have identified preterm infants with pulmonary hypertension that causes relevant oxygenation impairment with a high level of certainty. Given the fact that critical ill patients are usually provided with an arterial indwelling line to calculate relevant oxygenation metrics and regularly undergo full hemodynamics evaluation, this is a practicable definition. Our simplified definition of the pulmonary hypertension only based on the ratio of PAPs to systolic systemic pressure holds the risk of including healthy preterm infants with physiologically raised PAPs that has no relevant oxygenation consequence [31].

Another limitation of the study is that the groups are only moderately large, especially for preterm infants who had only a single complication. Another limitation is the exclusive inclusion of complications in the multiple regression analysis, which showed an effect on NT-proBNP levels in the univariate analysis and whose association with NT-proBNP levels has been demonstrated in a study-based manner. We thus aimed to strengthen the power of the multiple regression analysis, but this may have led to a false exclusion of factors.

## Conclusions

NT-proBNP levels in the first week of life appear to be strongly influenced by hsPDA, and the severity of PDA correlates with serum NT-proBNP levels. At 4 ± 1 weeks of age, BPD and BPD-related PH appear to be the main conditions associated with preterm birth that increase NT-proBNP levels.

## Data Availability

Derived data supporting the findings of this study are available from the corresponding author upon request.

## Abbreviations

BNP: Brain natriuretic peptide
BPD: Bronchopulmonary dysplasia
CRP: C-reactive protein
GA: Gestational age
hsPDA: Hemodynamically significant patent ductus arteriosus
IQR: Interquartile range
IVH: Intraventricular hemorrhage
La:Ao: Left atrial to aortic root ratio
NICU: Neonatal Intensive Care Unit
NT-proBNP: N-terminal pro-brain natriuretic peptide
PH: Pulmonary hypertension
ROP: Retinopathy of prematurity
SD: Standard deviation
